# Asymmetry of sonic hedgehog expression is necessary for early incisor tooth morphogenesis

**DOI:** 10.1242/dev.204409

**Published:** 2026-06-29

**Authors:** Ameera S. Haque, Masaki Hata, Kyle Marchuk, Pauline Marangoni, Jeremy B. A. Green, Ophir D. Klein

**Affiliations:** 1 Graduate Program in Oral and Craniofacial Sciences, School of Dentistry, University of California, San Francisco, San Francisco, CA 94143, USA.; 2 Program in Craniofacial Biology and Department of Orofacial Sciences, University of California, San Francisco, San Francisco, CA 94143, USA.; 3 Biological Imaging Development CoLab, University of California, San Francisco, San Francisco, CA 94143, USA.; 4 Department of Pediatrics, Cedars-Sinai Guerin Children’s, Los Angeles, CA 90048, USA.; 5 Centre for Craniofacial Regeneration and Biology, King’s College London, London SE1 9RT, UK.

**Keywords:** Tooth development, Shh signalling, Morphogenesis, Mouse

## Abstract

The dentition of mice comprises only two tooth types, molars and incisors. Despite similar epithelial-mesenchymal signalling interactions during development, their final morphologies differ dramatically. Both molars and incisors grow from a similarly symmetric epithelial organ bud starting at embryonic (E) day 12.5. By E13.5, they develop outgrowths known as cervical loops (CLs), which grow symmetrically in the molar but highly asymmetrically in the incisor. Prior work has shown epithelial *Shh* is required for the symmetric growth of molar CLs, but the cellular basis of incisor asymmetry is not known. Here, we report that differential proliferation in the epithelium and asymmetric distal *Shh* expression at E12.5 underlie asymmetric incisor morphogenesis. Inhibiting proliferation or *Shh* expression at this stage *ex vivo* resulted in a loss of asymmetry. Furthermore, symmetrizing the source of *Shh* at this stage by adding ectopic SHH protein *ex vivo* or expressing ectopic *Shh in vivo* similarly caused a loss of asymmetry. Together, these results demonstrate that spatial asymmetry of differential proliferation dependent on *Shh* expression plays a crucial role in the developing murine incisor.

## INTRODUCTION

Tooth development is a paradigmatic model for organ morphogenesis, showcasing how similar primordia give rise to diverse structures. Mice have two tooth types, molars and incisors, which, despite common origins, exhibit strikingly different morphologies tailored to their distinct functions. All ectodermal organs begin development as focal epithelial stratifications called placodes; in mice, tooth placodes form at embryonic day (E) 11.5 (Fig. 1A) (Jernvall and Thesleff, 2012) before thickening and invaginating into the underlying mesenchyme to form a symmetrical tooth bud (E12.5). Although both molar and incisor are morphologically symmetric at this stage, incisor patterning is already asymmetric: expression of the initiation knot (IK) signalling centre genes is distal in the incisor but central in the molar (Fig. 1A). Epithelial expansion, folding and formation of the enamel knot (EK) signalling centre follows, resulting in the late bud (E13.5), cap (E14.5) and bell (E15.5) stages. The IK and EK are clusters of non-dividing cells in the epithelium that express several families of signalling molecule (e.g. Shh, Fgf, Wnt, Tgfβ/Bmp and Eda) that are crucial in tooth formation. Molar and incisor morphology are visibly divergent by the cap stage, when two outgrowths, called cervical loops (CLs), flanking the EK grow downward symmetrically in the molar but asymmetrically in the incisor, which develops a longer distal (labial) CL and a short proximal (lingual) CL (Yu and Klein, 2020).

Sonic hedgehog (*Shh*) is an early marker of tooth development and is crucial to both incisor and molar initiation (Seppala et al., 2017). In *Shh* null mutants, the entire first branchial arch was lost, leading to severe, early defects prior to tooth morphogenesis (Chiang et al., 1996). However, epithelial-specific keratin 14 promoter-driven knockout (active from E11.5) of *Shh* resulted in asymmetric molars (with a highly malformed lingual CL) and incisors with ‘symmetrical’ CLs (at E18.5) (Dassule et al., 2000). *Ex vivo* experiments using Shh-blocking antibodies from E10.5 to E12.5 led to arrest of tooth morphogenesis at the placode stage (Cobourne et al., 2001). Explant studies in the molar showed that *Shh*-dependent cell intercalation is required for tooth bud invagination and that an Shh agonist can induce multiple invaginations resulting in aberrant bud morphology (Li et al., 2016; Panousopoulou and Green, 2016). Importantly for the present study, although Shh inhibition affected morphogenesis, it did not inhibit cell proliferation or tooth bud volume (Li et al., 2016). The above results show the indispensable role of *Shh* in tooth morphogenesis and underscore its significance in shaping dental structures. Such a role for Shh signalling has not been analysed in the incisor; however, *Shh* is asymmetrically expressed in the IK while its receptors and downstream effectors patched 1 (*Ptch1*), smoothened (*Smo*) and *Gli1* are expressed throughout the tooth germ (Hardcastle et al., 1998), suggesting it might play a role in determining the overall shape of the incisor.

Here, we characterize the cell behaviours that produce incisor asymmetry and show that it is caused by Shh-dependent asymmetry in epithelial cell proliferation. We further show, through loss- and gain-of-function Shh signalling mutants, the importance of asymmetric *Shh* expression in producing morphological asymmetry. Together, these findings elucidate a role for *Shh* in fine-tuning incisor tooth bud morphology that can enable morphogenetic steering of tooth shape.

## RESULTS AND DISCUSSION

### The emergence of asymmetry during murine incisor development is associated with a differential increase in basal epithelial cell numbers

Incisor asymmetry could arise through spatial differences in proliferation, cell size and/or shape, and/or delamination from the basal layer. To investigate which mechanism was responsible, we first computationally defined distal and proximal domains of the tooth bud as corresponding regions of low epithelial curvature (Fig. 1B) with no assumptions being made about whether the cell populations in these regions constitute lineage compartments. At E12.5, the incisor bud was symmetric by contour length, basal epithelial cell number (basal cells being defined as those in contact with the basement membrane) and cell density (cells per unit length of epithelium) (Fig. 1F-K). However, there was higher proliferation in the distal compared to proximal regions (Fig. 1L). By E13.5, the distal epithelium exceeded the proximal in both contour length and cell number (Fig. 1F,H), but there was no change or difference in cell density along the basement membrane (Fig. 1J). From E13.5 to E14.5, there was growth in the proximal region, as the proximal CL emerged, evidenced by an increase in contour length and cell number, while proximal cell density was also significantly increased (Fig. 1J). By E14.5, asymmetry was further increased by tighter packing of cells along the proximal epithelium compared to the distal. Differential distal proliferation continued at E13.5, but by E14.5, distal and proximal proliferation were not statistically different (Fig. 1L).

The mesenchyme starts forming a condensation near the EK signalling centre at E13.5, known as the dental papilla (Mammoto et al., 2015). We quantified this condensation by tracking mesenchymal cell density and proliferation from E12.5 to E14.5 (Fig. 1M-R). At E12.5, cell density was relatively even throughout the mesenchyme; by E13.5, we observed an increase in cell density in the proximal region. Additionally, in the proximal mesenchyme proliferation and cell density continued to dramatically increase in the dental papilla (region 4), as the tooth bud became increasingly asymmetric at E13.5-E14.5 (Fig. 1P-R).

Strong correlation (R^2^=0.9481) (Fig. 2A) between contour length and cell number suggested proliferation and/or differential delamination as the key mechanisms driving the incisor asymmetry visible by E13.5, rather than the significant differences in epithelial size or shape (cell width). The early difference in EdU incorporation and the correlation between contour length and cell number (R^2^=0.9481) together suggest that differential proliferation (rather than in cell delamination, size, or width) drives the incisor shape asymmetry seen at E13.5.

### Asymmetric incisor bud growth depends on early differential cell proliferation

To confirm the importance of proliferation timing in incisor asymmetry, we treated contralateral pairs of E12.0 and E13.0 incisor explants with a proliferation inhibitor (aphidicolin) or vehicle (DMSO), and quantified morphology after a 40 h culture (Fig. 2B). Complete inhibition of DNA synthesis was evidenced by the lack of EdU incorporation in aphidicolin-treated buds (Fig. 2C,F). Explants treated from E12.0 were smaller (lower bud depth) and more symmetric than controls (Fig. 2D,E), and failed to develop the proximal evagination associated with basal constriction adjacent to the EK (Yamada et al., 2019) seen in controls (Fig. 2C, left, arrowhead). Explants inhibited from E13.0, although smaller and shallower than controls (Fig. 2F,G), still formed CLs asymmetrically (Fig. 2H). Notably, since proliferation was inhibited after differential distal proliferation had been established at E13.0, residual growth from cells completing the cell cycle after the aphidicolin block could account for residual asymmetric CL formation (Fig. 2F-H). Together, these data show early differential proliferation is crucial to develop asymmetry in the incisor tooth bud. To understand the molecular control of this process, we further examined the role of Shh signalling.

### Incisor bud invagination asymmetry requires *Shh* signalling to the mesenchyme

Since *Shh* at E12.5 is an important morphogen in the molar (Dassule et al., 2000; Hardcastle et al., 1998; Li et al., 2016; Panousopoulou and Green, 2016) and is expressed exclusively from the IK (i.e. centrally in molar and distally in incisor), we hypothesized that *Shh* expression asymmetry drives asymmetric incisor morphogenesis. We first confirmed *Shh* was expressed in the IK at E12.5 in the incisor (Fig. 3A). As previously reported (Hardcastle et al., 1998; Hosoya et al., 2020), the downstream effectors *Ptch1* and *Gli1* are expressed in both the incisor epithelium and mesenchyme. There was asymmetry in *Ptch1* expression in the distal epithelium (region 3) compared to the proximal, indicating asymmetric Shh signalling activity at E12.5 (Fig. 3D).

We then inhibited Shh signalling in explants using the *Smo* antagonist cyclopamine (Fig. 3E). Treated explants had severely decreased *Ptch1* expression, and were shallower and wider compared to contralateral vehicle-treated controls (Fig. 3F-I), consistent with similar experiments previously reported in the molar (Li et al., 2016). This indicated that *Shh* is crucial for normal incisor bud invagination. Strikingly, the treated tooth buds were more symmetrical morphologically and lost the higher distal proliferation seen in controls, displaying a reversed ratio, with significantly elevated proximal proliferation instead (Fig. 3J).

A more pronounced phenotype was seen when mesenchymal *Shh* signal transduction was genetically abrogated *in vivo* by crossing an *Osr2*^*Cre/+*^ driver, which is expressed in the dental mesenchyme from E11.5 (Lan et al., 2007), with a *Smo*^*fl/fl*^ line (Fig. 3K) (Lan et al., 2001; Li et al., 2011; Long et al., 2001). We confirmed deletion of *Smo* in the proximal mesenchyme at E13.5 using *in situ* hybridization (Fig. 3L). The mutant buds were wider, shallower and more symmetric at E13.5 (Fig. 3O-Q) and at E14.5 (Fig. S1C-E), corroborating our *ex vivo* findings. Both *Ptch1* and *Gli1* were asymmetrically expressed (higher proximally) in E13.5 controls (Fig. 3N, Fig. S1A). This pattern was lost in *Osr2*^*Cre/+*^*;Smo*^*fl/fl*^ mutants and instead had higher *Ptch1* expression in the distal epithelium (Fig. 3K,N). Although mutant buds failed to progress beyond the symmetric bud morphology, they formed both an IK and EK (*Shh* expression) from E13.5 to E14.5 (Fig. 3K,M, Fig. S1B,G), indicating the phenotype was not due to developmental arrest. Furthermore, mutant buds continued to proliferate (EdU labelling), although mutants had similar levels of proliferation throughout, while control buds had higher proliferation in the distal epithelium and proximal mesenchyme (Fig. 3R, Fig. S1F). At E13.5, the pattern of epithelial cell density was consistent between mutants and controls (which persisted at E14.5), but there was significantly less proliferation and lower cell density in the proximal mesenchyme, and increased distal mesenchymal cell density in the mutants compared to controls (Fig. 3R-T). By E14.5, there was still lower cell density in the mutant proximal mesenchyme, but increased proliferation led to similar cell density compared to controls in the distal mesenchyme (Fig. S1F,I,J). These results reveal a key role for *Shh* in both incisor tooth bud invagination and asymmetry, but more particularly a crucial role for Shh-responsive mesenchyme. This is consistent with experiments showing that knockout of epithelial *Shh* responsiveness using *Krt14*^*Cre/+*^;*Smo*^*fl/fl*^ had no effect on overall molar or incisor morphology, although it did lead to enamel hypoplasia (Gritli-Linde et al., 2002).

### Ectopic *Shh* inhibits development of incisor asymmetry

To determine if the asymmetric *Shh* might be sufficient for asymmetric incisor morphogenesis, we tested the effects of ectopic Shh both *ex vivo* and *in vivo* (Fig. 4). We first provided an exogenous proximal source of SHH protein in the incisor tooth bud culture using an Affigel bead soaked in N-SHH protein or BSA vehicle as a control, and cultured for 30 h (Fig. 4A). This symmetrized the tooth bud shape without producing a significant defect in overall tooth bud depth or width (Fig. 4B-D). Overall proliferation was similar, but the distribution of proliferating cells changed: in controls, proliferation was higher in the distal compared to the proximal regions, while treated buds showed the reverse (Fig. 4F). This was like the effect of Shh inhibition (Fig. 3H). Taken together, with gain- and loss-of-function of Shh signalling producing similar outcomes, these results suggest that the distribution of *Shh* expression, rather than its level, produces the morphological asymmetry. They also demonstrate that localized Shh can steer morphogenesis.

To further validate these findings, we manipulated the distribution of Shh signalling *in vivo* using a conditional knock-in allele of *Shh* and the proximo-distally asymmetric *Osr2*^*Cre/+*^ allele to drive proximal mesenchymal *Shh* expression (Castillo et al., 2014) (Figs 3L, 4G). As intended, the *Osr2*^*Cre/+*^;*Shh*^*YFPcKI/+*^ compound mutant ectopically expressed *Shh* in the proximal mesenchyme of the incisor tooth bud from E11.5 (Fig. 3L). At E13.5 the mutant buds were significantly smaller and more symmetric than controls (Fig. 4H-J). In the mutant at E13.5, *Shh* was expressed throughout the tissue, but also more highly in the distal epithelium, suggesting the tooth bud did form an IK, but failed to form the subsequent EK (Fig. 4N). As expected, both *Ptch1* and *Gli1* activity was greater in all regions of the mutant compared to controls, and greatest in the proximal mesenchyme (Fig. 4O,P). Mutants also lacked the higher proliferation and cell density in the distal epithelium and proximal mesenchyme that was seen in controls (Fig. 4K-M). Like the loss of Shh signalling, ectopic *Shh* also disrupted mesenchymal condensation, with significantly lower cell density in the proximal mesenchyme compared to controls (Fig. 3T). By E15.5, mutant buds were wider and shallower than at E13.5, and remained symmetric (Fig. S2E,F). *Ptch1* expression at E15.5 was asymmetric in the mutants with elevated expression in the proximal mesenchyme. Although *Shh* expression in the mutants was elevated in all regions compared to the controls, the mutant tooth buds failed to form an EK (Fig. S2C,D). At E15.5, cell density was greater in the mutant distal mesenchyme compared to controls (Fig. S2H,I). Proliferation in both the control and mutant tooth buds was asymmetric, with higher proliferation in the proximal epithelium and mesenchyme compared to the respective distal compartments (Fig. S2B).

Together, our results indicate that Shh signalling not only facilitates initial bud invagination but also contributes to the development of incisor asymmetry. We found that proximodistal incisor asymmetry is primarily based on larger numbers of similarly sized basal cells in the distal versus proximal epithelium, and this difference is predominantly or entirely due to differential epithelial proliferation. We showed that the asymmetry depends on asymmetrically localised Shh signalling. However, although Shh has previously been described as a mitogen in the context of tooth development, we found – consistent with previous careful quantification of Shh signalling perturbation (Li et al., 2016) – that this is not the case: Shh affects the distribution of proliferation, but not its overall level. Additionally, since both gain and loss of Shh function symmetrized the incisor tooth bud shape, these results revealed it is the distribution rather than the mere level of Shh that is crucial.

How, then, does Shh generate asymmetry? We show that the mesenchyme plays a crucial role, since conditional removal of mesenchymal-specific Shh responsiveness also symmetrizes the structure. The mechanism of this Shh-dependent mesenchyme involvement remains unclear. One possibility is that Shh produced by the IK triggers local production in the distal mesenchyme of a epithelium-directed mitogen. There could also be mechanical effects: we observed lower cell density in distal mesenchyme (Fig. 1Q) suggesting that Shh from the IK could inhibit mesenchymal condensation distally, allowing elevated distal epithelial expansion. In both the *Osr2*^*Cre/+*^;*Smo*^*fl/fl*^, where *Shh* does localize but to an expanded EK region, and *Osr2*^*Cre/+*^;*Shh*^*YFPcKI/+*^ mutants, where the EK does not form, the buds remain symmetrical and fail to form the characteristic proximal epithelial evagination from E13.5 onwards. In both loss and gain of Shh signalling mutants, we saw a loss of this mesenchymal condensation pattern – decreased cell density in the proximal mesenchyme as opposed to increased cell density in the distal mesenchyme in the mutants compared to controls – which further implies the localization of *Shh* expression and activity is crucial to this system (Fig. S3).

Finally, our results represent an attempt at what might be termed ‘morphogenetic steering’ – using externally applied signals to redirect morphogenesis in a defined way. They reveal that, while this is possible, we are far from untangling direct effects on morphogenetic cell behaviours from morphogen-driven repatterning. Prior work has shown a potential to bioengineer the tooth germ from dissociated epithelial and mesenchymal cells to form correct tooth structures (i.e. ameloblasts, enamel, etc.) but not necessarily recapitulating natural morphology (Nakao et al., 2007). Our work provides a potential paradigm for the manipulation of morphology, with its potential for tuning and optimizing *in vitro* models.

## MATERIALS AND METHODS

### Mouse genetics and embryo staging

*Ex vivo* experiments were conducted using *Krt14*^*Cre/+*^;*Rosa26*^*mTmG/mTmG*^ mice (Dassule et al., 2000; Muzumdar et al., 2007). Embryonic stages were calculated from the date of vaginal plug at E0.5, and wet body weight was checked to ensure consistency (Peterka et al., 2002). Embryos were then fixed whole or dissected for explant culture. For cell proliferation experiments, pregnant dams were injected with 25 μg/g EdU for 1 h prior to euthanasia. Mutant lines were crosses of published *Osr2*^*Cre*^*/*^*+*^, *Smo*^*fl/fl*^, and *Shh*^*YFPcKI/+*^ strains maintained on mixed background and interbred via *Osr2*^*Cre/+*^ breeders from both *Osr2*^*Cre/+*^;*Smo*^*fl/fl*^ and *Osr2*^*Cre/+*^;*Shh*^*YFPcKI/+*^ over ~16 generations (Castillo et al., 2014; Lan et al., 2001; Long et al., 2001). Experiments performed in this study were pre-approved by the UCSF Institutional Animal Care and Use Committee.

### Explant culture and treatments

Explant slice culture followed the methods of Alfaqeeh and Tucker (2013), with minor modifications: sagittal tissue slices containing the incisor tooth buds were manually cut from E12.5 or E13.5 mandibles then placed in 15 μl of 50% laminin (Cultrex) on a 0.05 μm PET filter (Sterlitech) floating on modified F12 medium (Dulbecco) supplemented with N2, B27, fetal bovine serum and antibiotic-antimycotic (Thermo Fisher). Inhibition experiments included 5 μM aphidicolin (CST 32774S), 20 μM cyclopamine (ab120392) or DMSO vehicle. The bead protocol was adapted from Tiecke and Tickle (2007): Affi-gel Blue beads (BioRad 1537302) were incubated with 500 ng/μl N-SHH (R&D Systems 461-SH-025) in 0.1% BSA or vehicle alone for 1 h at 37°C, then placed in the explant using fine forceps. For all cultures, 5 μg/ml EdU was added for 1 h before fixation. The vestibular lamina (vl), although fragile and sometimes lost during subsequent handling, was always visible as a distinct structure at the time of explant, and was used to distinguish the incisor from the vl and to orient the tissue.

### Tissue processing, immunostaining and RNAscope

Whole embryos and explants were fixed in 4% PFA overnight at 4°C with shaking, washed with PBS (pH 7.4), cleared using CUBIC-L (Susaki et al., 2014) for 48 h at 37°C with shaking, then washed several times with PBS before staining. Mandibles from fixed embryos were embedded in 4% low melt agarose, sectioned using a vibratome at 120 μm, and cleared using CUBIC-L before staining (Matsumoto et al., 2019) using 1:500 anti-GFP (Aves GFP-1020) and/or 1:200 anti-E-cadherin (BD 610181) primary antibodies, followed by Alexa Fluor 647 donkey secondary antibodies. EdU staining was carried out with the Click-iT EdU kit (Invitrogen C10640).

For RNAscope, embryos were fixed 24 h in 4% PFA, equilibrated with 30% sucrose and then embedded in 30% sucrose:OCT (cryo-embedding medium) prior to sectioning using a cryotome to obtain 10 μm slices. Slides were processed following the standard protocol (available online) with some adjustments. Slides were baked at 60°C for at least 90 min, post-fixed for 15 min in 4% PFA at 4°C, followed by dehydration and 5 min H_2_O_2_ treatment, 5 min antigen retrieval, and 30 min incubation with protease III. The probes used were *Shh* (no. 314361, ACDBio), *Ptch1* (no. 402811, ACDBio) and *Gli1* (no. 311001, ACDBio). Sections were co-stained for E-cadherin, EdU and DAPI following the RNAscope.

### Imaging and image analysis

All tissues were imaged using a Zeiss LSM900 confocal microscope with a 25x Plan NeoFluar objective (Zen software v. 3.4.91.00000). Distal and proximal regions were defined using a MATLAB-based software that we have named ‘multi-use application for reporting general and region-specific integral tissue attributes’ (i.e. MARGARITA) to calculate the radius of curvature (ROC). Images of tooth bud epithelia (labelled by reporter fluorescence proteins in *Krt14*^*Cre/+;*^*Rosa26*^*mTmG/mTmG*^ embryos or by immunofluorescence staining of E-cadherin) can be input into this program, which will then render a detailed 3D surface object of the tooth bud tissue. For each point along this rendered surface, MARGARITA calculates the ROC by fitting a circle to three adjacent points. The resulting output is the length of the radius of the fitted circle; a smaller ROC indicates a curved surface, while a larger ROC indicates a flatter surface. For all analyses in this study, the endpoints of the distal (cyan) and proximal (yellow) regions were identified by regions of high curvature of the tooth bud neck and CLs; color-coded contours indicate the measured regions (as shown in Fig. 1B).

Tooth bud width and depth, and distal and proximal contour lengths were measured using Fiji (Schindelin et al., 2012). Width was measured as the distance between the distal and proximal bud neck. Depth was measured from the bud neck to the tip of the tooth bud at its deepest point. Cells in each region were counted manually in the optical slice containing their maximum cross-sectional area to avoid grazing artifacts. Only basal epithelial cells (in contact with the basal lamina) were counted. All mesenchymal cells in regions identified in Fig. 1M-R were counted and cell density per 100 μm^2^ was calculated. To measure proliferation, EdU-positive nuclei were counted in the respective regions to calculate EdU index [(number of Edu^+^ cells/total cells)x100]. For RNAscope signal quantification fluorescence pixel intensity was summed in the indicated regions of interest using Fiji then divided by region area. E13.5 mutant comparisons were run in parallel, sharing the same *Osr2*^*Cre/+*^ control group (Figs 3K and 4G).

### Statistics

Graphs were plotted using GraphPad Prism. Error bars indicate the standard error of the mean (s.e.m.); individual data points are plotted. Statistical analysis of single comparisons of control versus mutant tissues and control versus treated explants were measured using unpaired and Student’s *t*-test, respectively. One-way analysis of variance (ANOVA) and Tukey’s post-hoc test for multiple comparisons was used to compare cell density, EdU index or gene expression across different regions (Figs 1M-R, 2B). A two-way ANOVA with Tukey correction for multiple comparisons was used to compare distal versus proximal metrics over time or in different compartments (i.e. epithelium and mesenchyme). All statistical comparisons were two-tailed.

## Supplementary Material

Supplementary Information file

## Figures and Tables

**Fig. 1.**
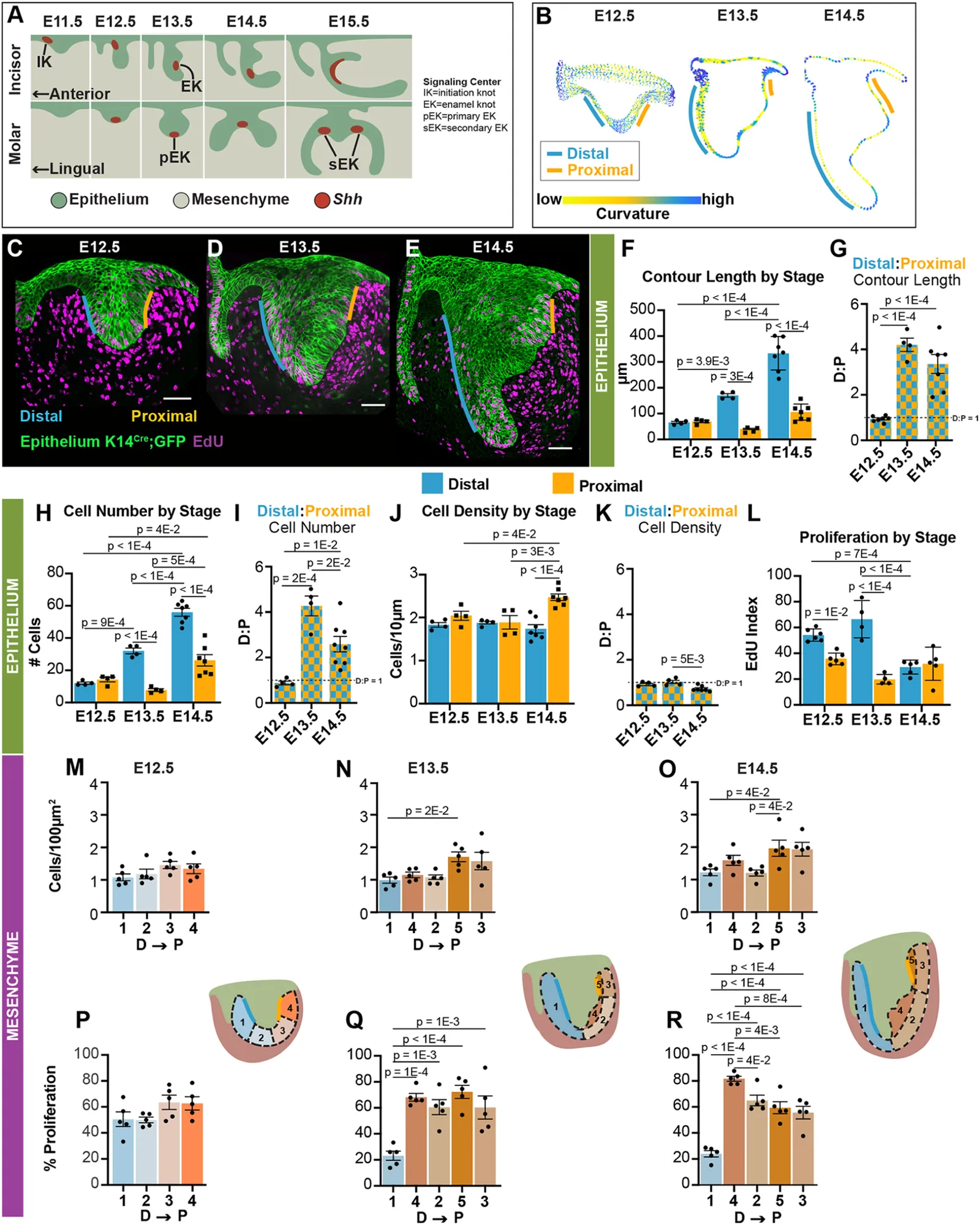
Differential epithelial cell number, proliferation and mesenchymal cell density underlie E12.5–14.5 incisor contour length asymmetry. (A) Schematic of early incisor and molar development showing the changes in epithelial tooth bud morphology (green) and *Shh*-expressing signalling centre locations (red) from embryonic day (E) 11.5 to E15.5. (B) Example heatmaps of bud epithelium curvature of E12.5-E14.5 tooth of the kind generated for each image in this study to define distal and proximal domains. (C-E) Micrographs corresponding to B from sagittal sections of *Krt14*^*Cre/+*^;*Rosa26*^*mTmG/mTmG*^ incisors showing membrane GFP-positive epithelium (green), proliferation (EdU incorporation, magenta), and curvature-defined distal (blue) and proximal (gold) domains. (F,H,J,L) Epithelial contour lengths (F; μm), cell numbers (H), cell densities (J; cells/10 μm) and EdU index values overtime (L; E12.5-E14.5). (G,I,K) Distal:proximal (D:P) ratios of contour length (G), cell number (I) and cell density (K) over time. (M-R) Mesenchymal cell density (M-O; cells/100 μm^2^) and proliferation (P-R; % EdU-positive cells) at each stage. Regions measured are shown in colour-coded schematics. Only statistically significant (*P*<0.05) *P*-values are displayed. Scale bars: 50 μm. Individual datapoints are shown (*n*≥4 for each timepoint). Data are mean±s.e.m.

**Fig. 2.**
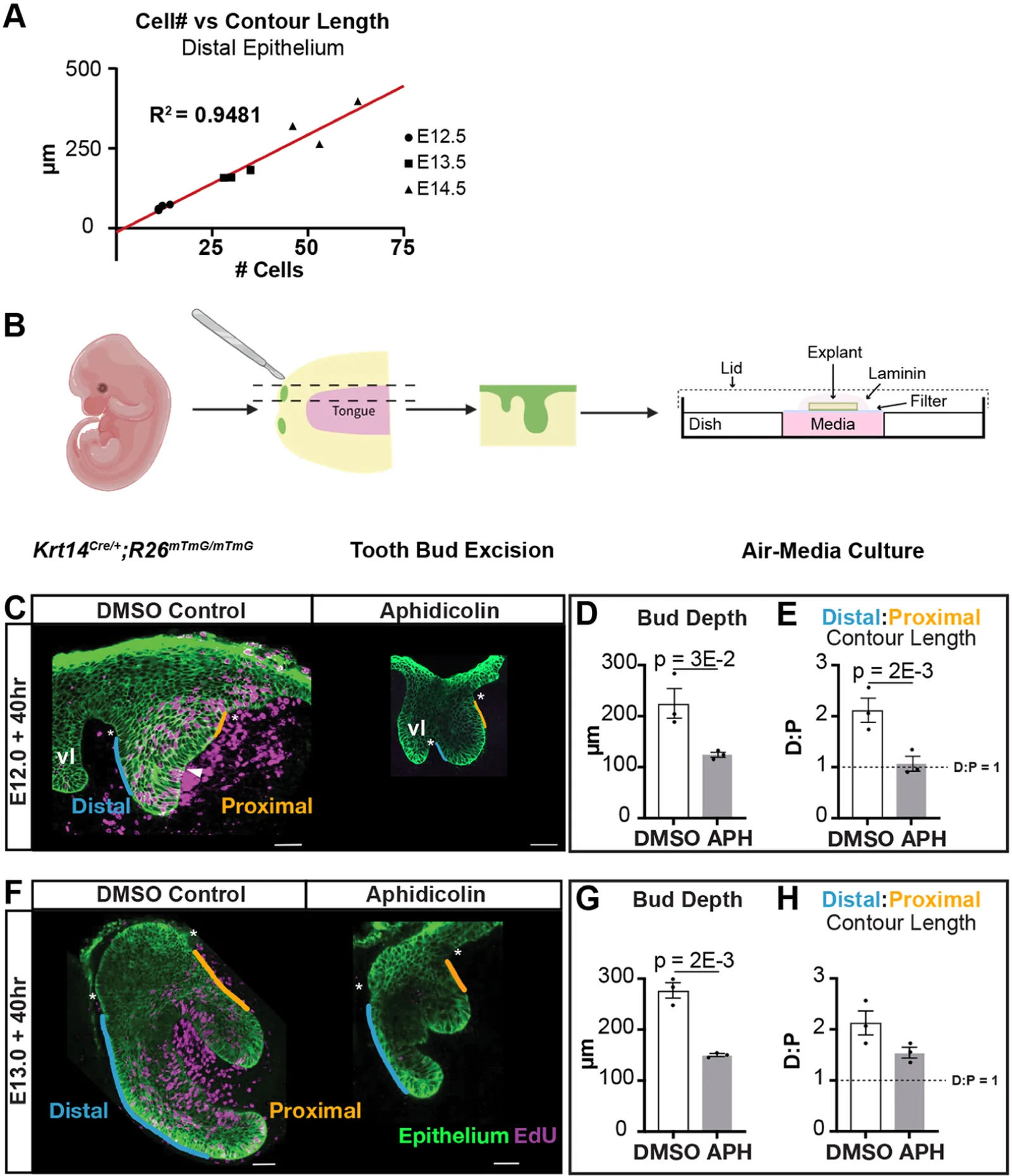
Cell proliferation differences are sufficient to explain incisor distal-proximal asymmetry. (A) Scatter plot of distal epithelial cell number versus contour length from wild-type E12.5-E14.5 *Krt14*^*Cre/+*^;*Rosa26*^*mTmG/mTmG*^ tooth buds. Simple regression line shown in red and R^2^ coefficient reported numerically. (B,C,F) Contralateral pairs of sagittal *Krt14*^*Cre/+*^; *Rosa26*^*mTmG/mTmG*^ incisor tooth bud explants were dissected, manually sliced and cultured from E12.0 (*n*=6) (C) or E13.0 (*n*=4) (F) in an air-medium interface with vehicle (DMSO) or proliferation inhibitor (aphidicolin) for 40 h. EdU was added to the culture media for the last hour then fixed, cleared, stained and imaged. Curvature-defined distal (blue) and proximal (gold) domains are indicated. (D,E,G,H) Bud depth (D,G), measured from the neck to the tip of the bud, and D:P ratio (E,H), quantified from paired vehicle and drug-treated samples. All metrics were compared with a two-tailed, paired *t*-test with *P*-values displayed numerically. vl, vestibular loop. Individual datapoints are shown. Data are mean±s.e.m. Asterisks indicate tooth bud neck; arrowhead indicates evagination. Scale bars: 50 μm.

**Fig. 3.**
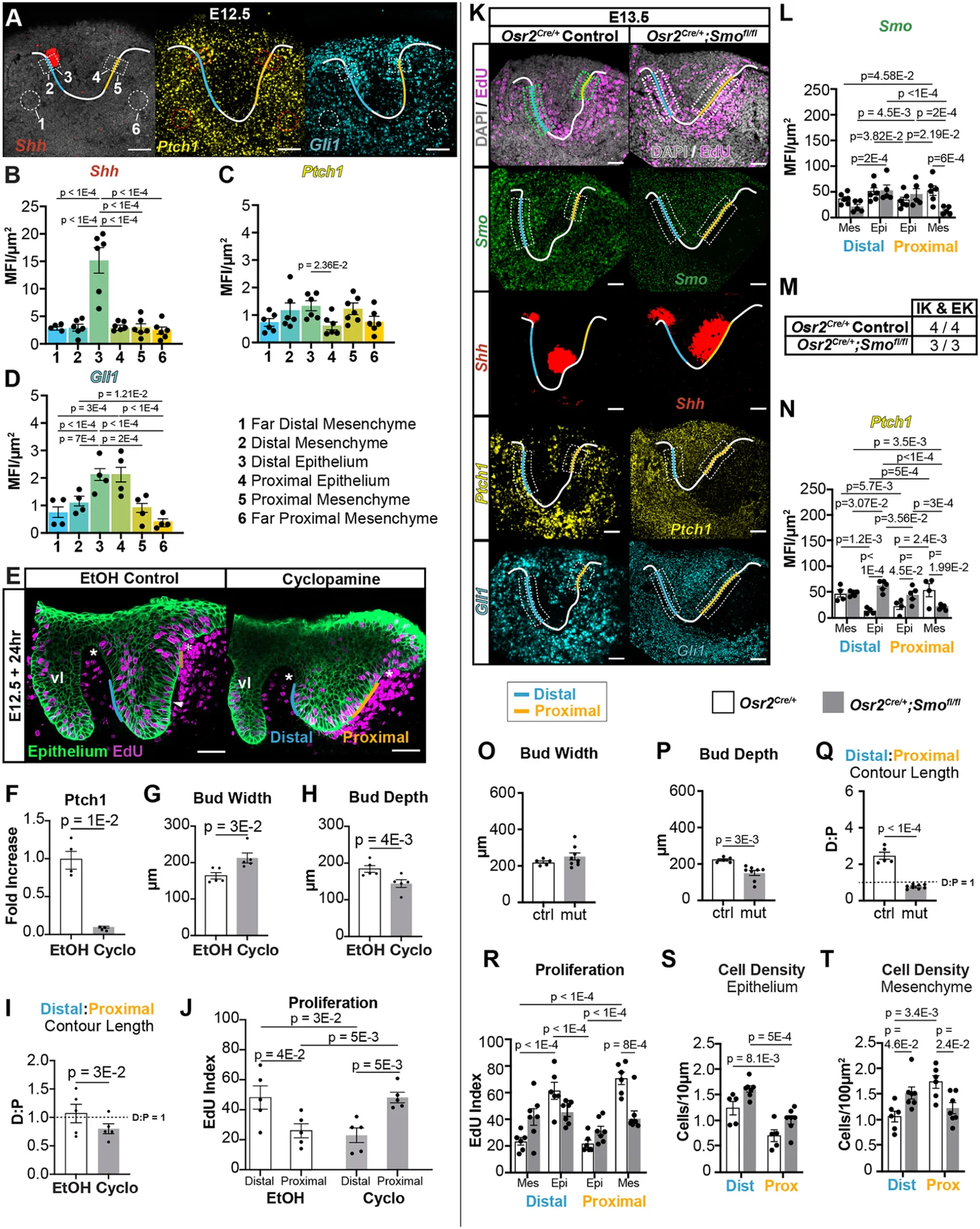
Inhibiting Shh signalling *ex vivo* and *in vivo* prevents proper invagination and asymmetric morphogenesis. (A) Expression of *Shh* (red), *Ptch1* (bright yellow) and *Gli1* (cyan) in the E12.5 incisor tooth bud, as detected by RNAscope *in situ* hybridization. Curvature-defined distal (blue) and proximal (gold) domains are indicated. (B-D) Expression quantified as integrated fluorescence pixel intensity divided by area from regions 1–6 indicated by dashed lines in A. Significance was determined using one-way ANOVA followed by pairwise Tukey testing. (E) Contralateral pairs of sagittal slice explants of incisor tooth buds from E12.5 *Krt14*^*Cre/+*^;*Rosa2S*^*mTmG/mTmG*^ *+/−Shh* inhibitor (cyclopamine) or vehicle (ethanol) cultured for 24 h with EdU added 1 h prior to fixation, clearing and staining. (F-J) *Ptch1* expression (qPCR), tooth bud width, depth and D:P ratios of contour length and EdU index (*n*=6) of treated and control-treated explants. Metrics are compared using a two-tailed, paired *t*-test and one-way ANOVA. (K) RNAscope *in situ* hybridization images of E13.5 incisor tooth buds from *Osr2*^*Cre/+*^;*Smo*^*fl/fl*^ and *Osr2*^*Cre/+*^ littermate controls (*n*=5 pairs, shared with Fig. 4G) for the indicated genes. (L,N,R-T) Dashed lines in K indicate epithelial and mesenchymal regions measured to quantify (L) *Smo* (green), (N) *Ptch1* (bright yellow) gene expression, and distal-proximal (R) EdU index and (S,T) cell density. All metrics are compared using a two-way ANOVA with Tukey multiple comparisons, with *P*-values displayed numerically. (M) The numbers of IK and EK signalling centres in control and mutant buds marked by *Shh* (red in K). (O-Q) Tooth bud width (O), depth (P) and D:P ratio (Q) quantified and compared using a two-tailed, unpaired *t*-test. Individual datapoints are shown. Data are mean±s.e.m. epi, epithelium; mes, mesenchyme; vl, vestibular loop. Asterisks indicate tooth bud neck. Scale bars: 50 μm.

**Fig. 4.**
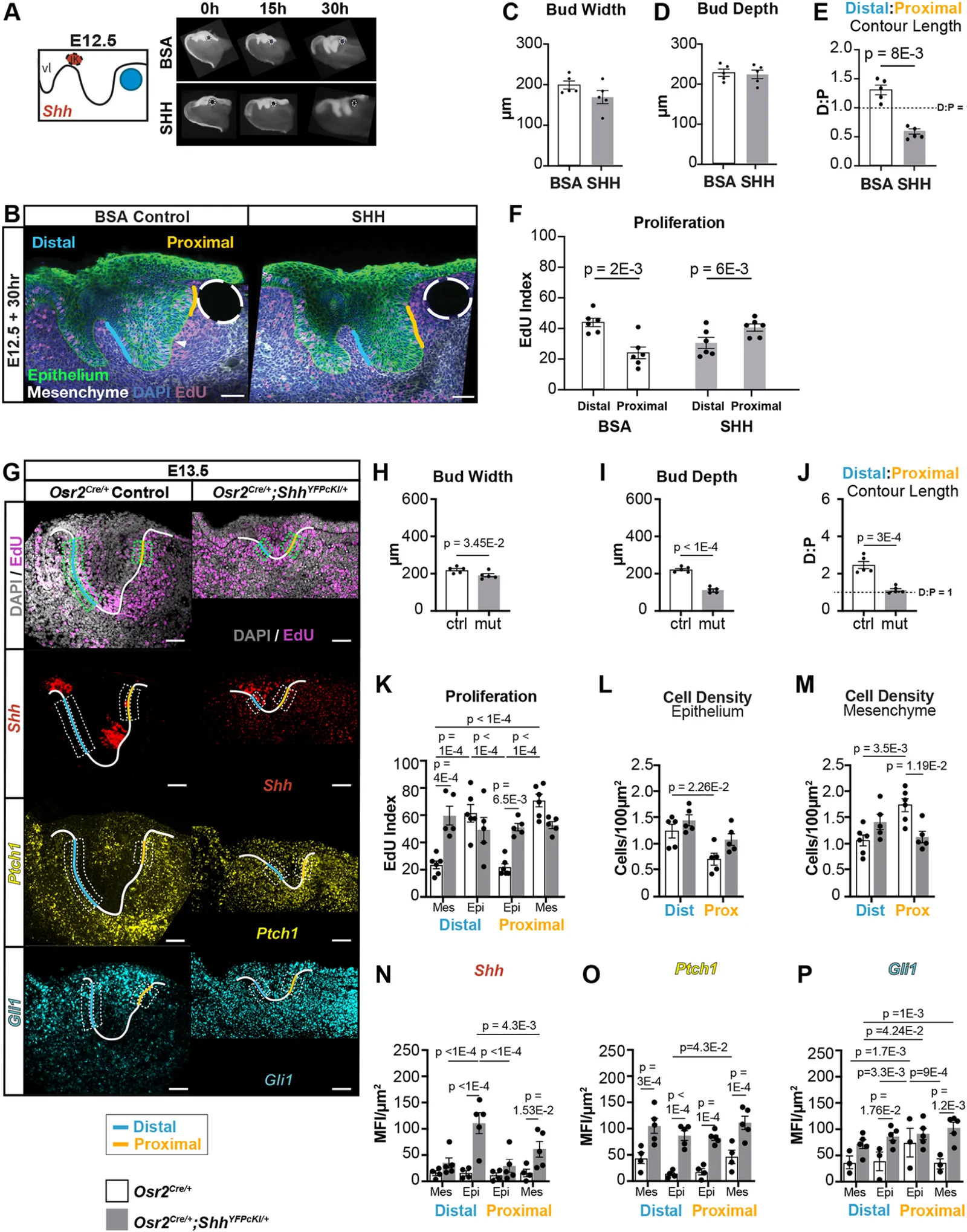
Ectopic SHH protein *ex vivo* and *Shh* expression *in vivo* effectively symmetrize incisor bud morphology and disrupt development of distal-proximal asymmetry. (A) Contralateral pairs of sagittal slices of incisor tooth buds from E12.5 *Krt14*^*Cre/+*^;*Rosa26*^*mTmG/mTmG*^ mandibles were manually dissected and cultured with a proximally placed Affi-gel bead soaked in N-SHH or BSA (vehicle) protein in an air-media interface system. (B) Explants were cultured for 30 h with EdU added for the last hour prior to fixation, clearing and staining (epithelial cell membrane, green; mesenchymal cell membrane, white; bead, dashed white circle; DAPI, blue; EdU+, purple). Curvature-defined distal (blue) and proximal (gold) domains are indicated. (C-F) Tooth bud width (C), depth (D), D:P ratio of contour length (E) and EdU index (F) quantified (*n*=5). Metrics were compared using a two-tailed, paired *t*-test and two-way ANOVA, with *P*-values displayed numerically. (G) Ectopic *Shh* expressed *in vivo* using the *Osr2*^*Cre/+*^;*Shh*^*YFPcKI/+*^ mouse line. Sagittal sections of fixed 13.5 incisor tooth buds from *Osr2*^*Cre/+*^;*Shh*^*YFPcKI/+*^ and *Osr2*^*Cre/+*^ (*n*=5 pairs) littermate controls (control dataset shared with Fig. 3K). (H-J) Tooth bud width (H), depth (I) and D:P ratio (J) of contour length quantified and compared using an unpaired *t*-test. Dashed lines in G indicate regions where (K) EdU index, (L,M) epithelial and mesenchymal cell density, (N) *Shh* (red), (O), *Ptch1* (yellow), and (P) *Gli1* (cyan) gene expression were quantified. All metrics were compared using two-way ANOVA with Tukey multiple comparisons, with *P*-values displayed numerically. Individual datapoints are shown. Data are mean±s.e.m. epi, epithelium; mes, mesenchyme. Scale bars: 50 μm.

## Data Availability

The MARGARITA code has been deposited in GitHub (https://github.com/kdmarchuk/cell_isolator_shape_analysis.git). All other relevant data and details of resources can be found within the article and its supplementary information.
