# Supplementary material for: Asymmetry of sonic hedgehog expression is necessary for early incisor tooth morphogenesis
Source: Development. Author manuscript; Available in PMC 2026 Oct 20. (PMC13682700; doi:10.1242/dev.204409)
Supplement: Supplementary file 1 [file NIHMS2207634-supplement-Supplementary_Information_file.pdf]

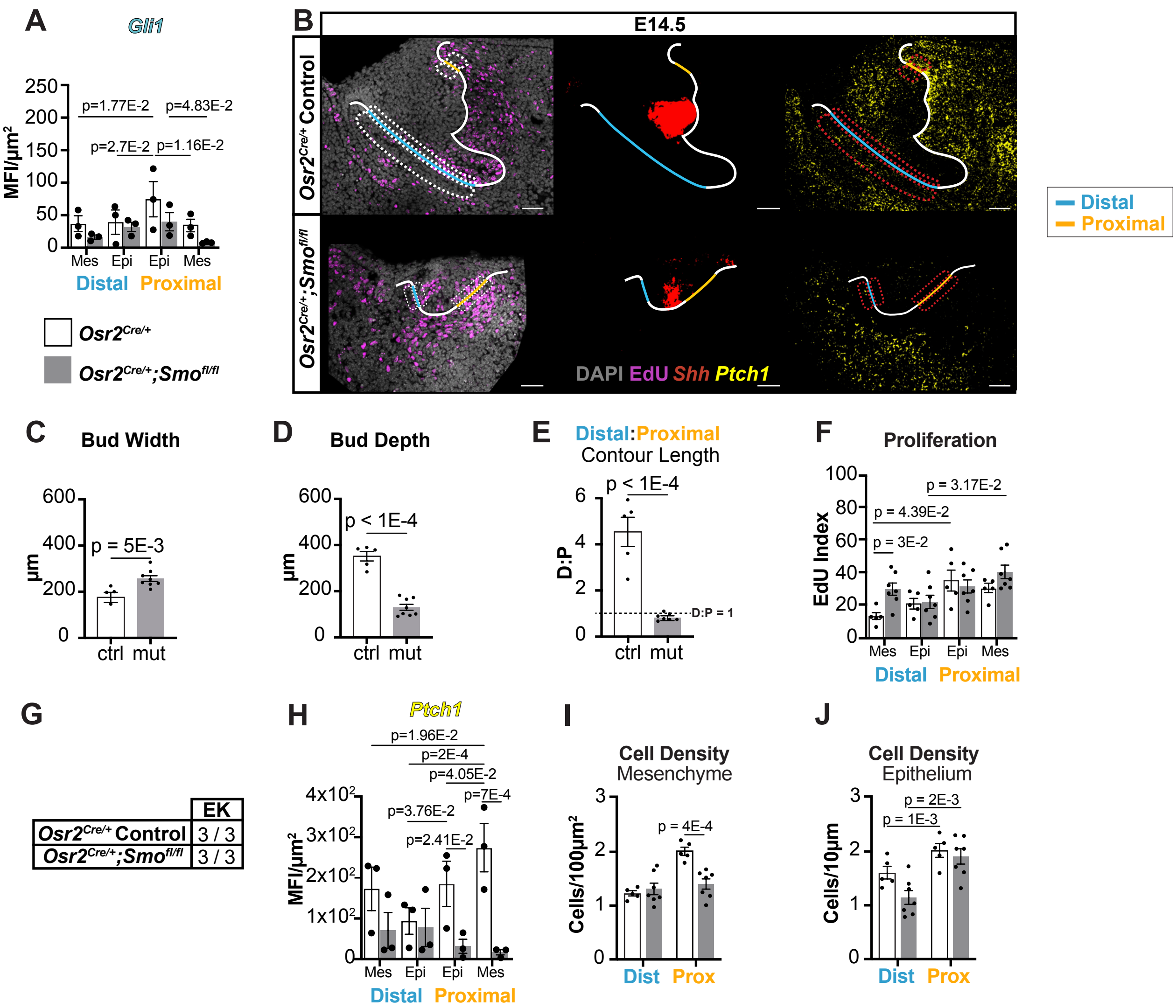

Fig. S1. *In vivo* inhibition of Shh at E13.5-14.5 inhibits asymmetry at the tissue and cell level. (A)*In vivo* mesenchymal *Shh* signaling ablated using *Osr2*$^{Cre/+}$*;Smo*$^{fl/fl}$ mouse line at E13.5 (Fig. 3K). *Gli* (cyan) gene expression levels at E13.5 between *Osr2*$^{Cre/+}$*;Smo*$^{fl/fl}$ and *Osr2*$^{Cre/+}$ littermate controls (n=3 pairs, shared with Fig. 4G run in parallel) quantified and compared by two-way ANOVA. (B) Sagittal sections of fixed E14.5 incisor tooth buds from *Osr2*$^{Cre/+}$*;Smo*$^{fl/fl}$ (n=7) and *Osr2*$^{Cre/+}$ littermate controls (n=5). Tooth bud width, depth, and D:P ratio quantified and compared by unpaired t-test in (C-E). (G) *Shh* (red) gene expression marks enamel knot (EK) signaling center, and presence of EK in control and mutant buds recorded in a table. Dashed lines indicate epithelial and mesenchymal regions measured to quantify distal proximal EdU index, *Ptch1* (bright yellow) gene expression, and cell density quantified in (F, H-J). All metrics compared by two-way ANOVA with Tukey multiple comparisons and statistically significant p-values are displayed numerically. *Osr2*$^{Cre/+}$=white bars, *Osr2*$^{Cre/+}$*;Smo*$^{fl/fl}$=grey bars, dashed lines=regions of interest, cyan=*Gli1*, EdU+=magenta, DAPI=gray, *Shh*=red, *Ptch1*=bright yellow, distal=blue, proximal=gold, scale bars=50 µm.

Development • Supplementary information

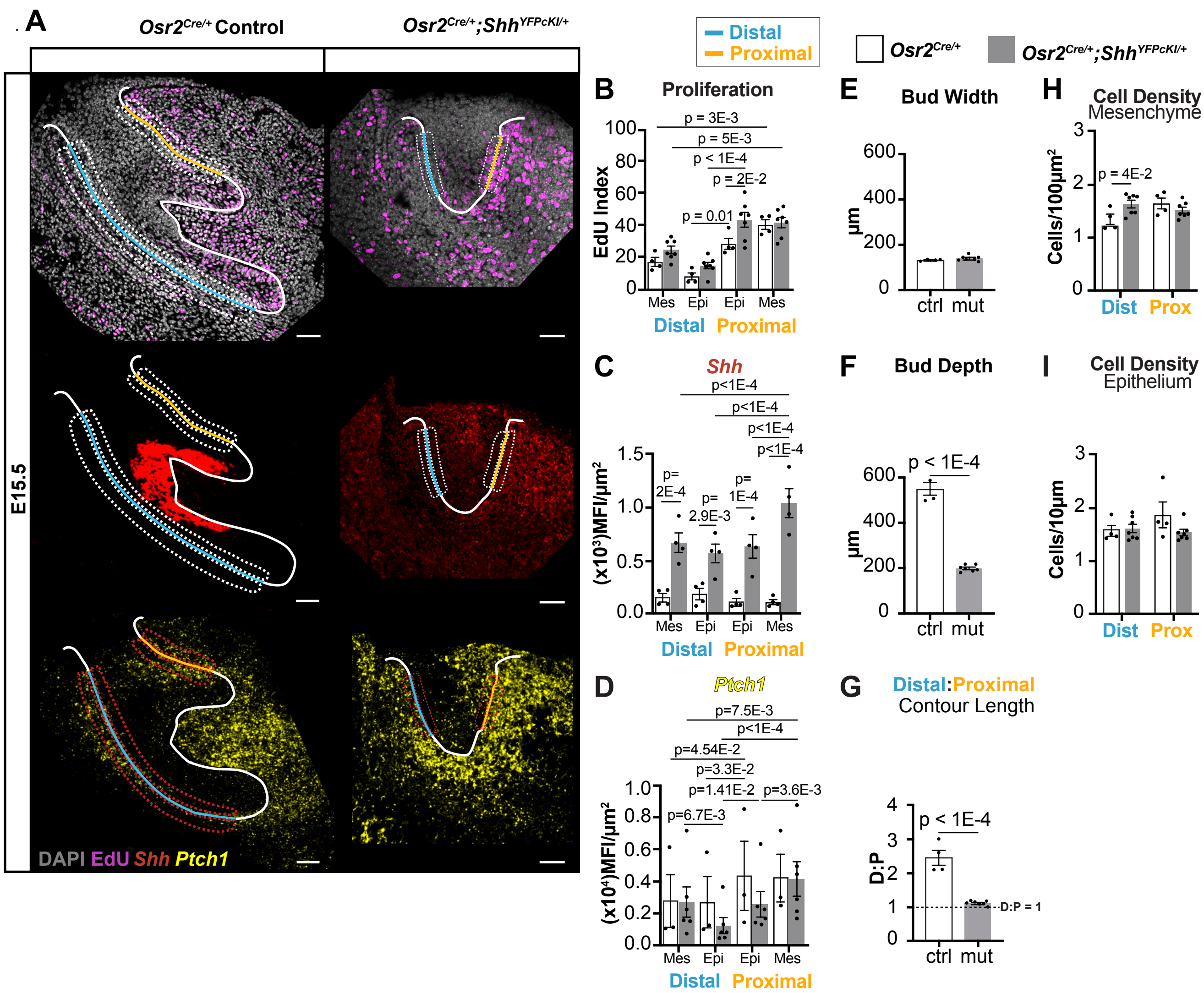

Fig. S2. Ectopic *Shh* expression at E15.5 *in vivo* shows loss of asymmetry. (A) Ectopic *Shh* expressed *in vivo* using the *Osr2*$^{Cre/+}$*;Shh*$^{YFPcKI/+}$ mouse line. Sagittal sections of fixed 15.5 incisor tooth buds from *Osr2*$^{Cre/+}$*;Shh*$^{YFPcKI/+}$ (n=6) and *Osr2*$^{Cre/+}$ littermate controls (n=4 pairs). (B-D, H-I) Quantification of EdU index, *Shh* (red) and *Ptch1* (bright yellow) gene expression, mesenchymal and epithelial cell density measured in regions outlined (dashed lines). All metrics compared by two-way ANOVA with Tukey multiple comparisons and significant p-values are displayed numerically. (E-G) Tooth bud width, depth, and D:P ratio of contour length quantified and compared by unpaired t-test. All statistically significant p-values are displayed numerically. *Osr2*$^{Cre/+}$=white bars, *Osr2*$^{Cre/+}$*;Shh*$^{YFPcKI/+}$=grey bars, region of interest=dashed, distal=blue, proximal=gold, EdU+=magenta, DAPI=gray, *Shh*=red, *Ptch1*=bright yellow, epi=epithelium, mes=mesenchyme, scale bars=50 µm.

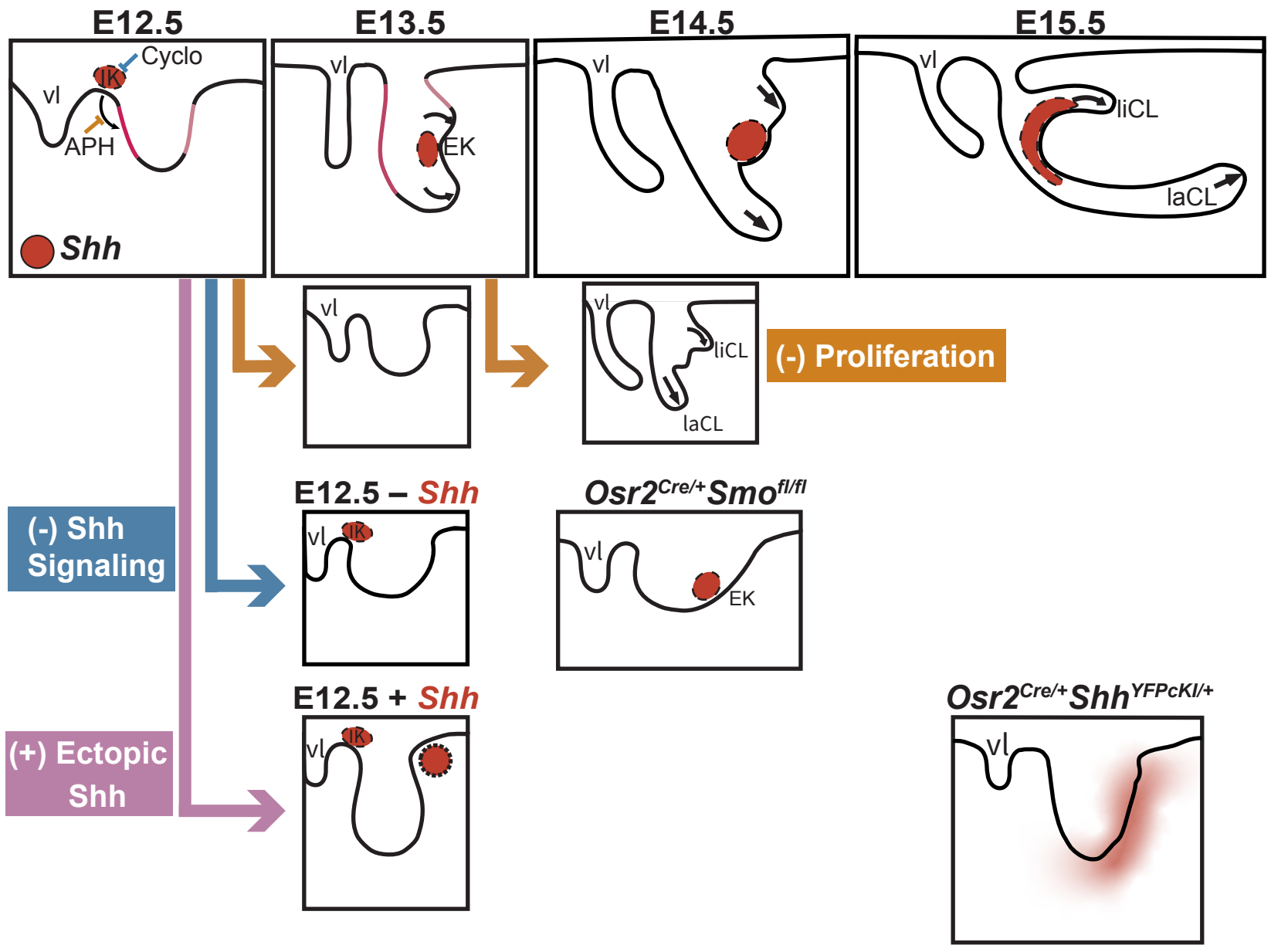

Fig. S3. Summary of early asymmetric development of the mouse incisor. (A) Model proposing increased signaling to the distal tooth bud from distal initiation knot (IK) signaling center during development from symmetric E12.5 (bud) to asymmetric E13.5 (late bud stage). (B) Inhibition of proliferation at E12.5 prevents growth beyond bud stage, and inhibition at E13.5 results in a smaller tooth bud that still forms asymmetric cervical loops and cap morphology. (C) Inhibition of Shh signaling both *ex* vivo, at E12.5+24h, and *in vivo*, at E14.5, inhibits bud invagination but tooth bud development still progresses as evidenced by the formation of the *Shh*-expressing enamel knot (EK) *in vivo*. (D) Ectopic Shh signaling both *ex vivo*, using SHH-soaked beads, and *in vivo*, in *Osr2*$^{Cre/+}$;*Shh*$^{YFPcKI/+}$ mutant embryos, prevents incisor asymmetry. Additionally, mutant incisors fail to form both cervical loops and the EK at E15.5.
